# Temporal variation of genetic composition in Atlantic salmon populations from the Western White Sea Basin: influence of anthropogenic factors?

**DOI:** 10.1186/1471-2156-14-88

**Published:** 2013-09-23

**Authors:** Mikhail Yu Ozerov, Alexey E Veselov, Jaakko Lumme, Craig R Primmer

**Affiliations:** 1 Department of Biology, Division of Genetics and Physiology, University of Turku, 20014 Turku, Finland; 2 Institute of Biology, Karelian Research Centre RAS, Pushkinskaya 11, 185610 Petrozavodsk, Russia; 3 Department of Biology, University of Oulu, P.O. Box 3000, 90014 Oulu, Finland; 4 Kevo Subarctic Research Institute, University of Turku, Turku 20014, Finland

**Keywords:** Atlantic salmon, Temporal variation, Genetic diversity, Genetic structure, Fishing pressure

## Abstract

**Background:**

Studies of the temporal patterns of population genetic structure assist in evaluating the consequences of demographic and environmental changes on population stability and persistence. In this study, we evaluated the level of temporal genetic variation in 16 anadromous and 2 freshwater salmon populations from the Western White Sea Basin (Russia) using samples collected between 1995 and 2008. To assess whether the genetic stability was affected by human activity, we also evaluated the effect of fishing pressure on the temporal genetic variation in this region.

**Results:**

We found that the genetic structure of salmon populations in this region was relatively stable over a period of 1.5 to 2.5 generations. However, the level of temporal variation varied among geographical regions: anadromous salmon of the Kola Peninsula exhibited a higher stability compared to that of the anadromous and freshwater salmon from the Karelian White Sea coast. This discrepancy was most likely attributed to the higher census, and therefore effective, population sizes of the populations inhabiting the rivers of the Kola Peninsula compared to salmon of the Karelian White Sea coast. Importantly, changes in the genetic diversity observed in a few anadromous populations were best explained by the increased level of fishing pressure in these populations rather than environmental variation or the negative effects of hatchery escapees. The observed population genetic patterns of isolation by distance remained consistent among earlier and more recent samples, which support the stability of the genetic structure over the period studied.

**Conclusions:**

Given the increasing level of fishing pressure in the Western White Sea Basin and the higher level of temporal variation in populations exhibiting small census and effective population sizes, further genetic monitoring in this region is recommended, particularly on populations from the Karelian rivers.

## Background

Most empirical studies of genetic structure in natural populations use a single sampling time point, assuming that the genetic pattern is stable over time [1]. However, temporal fluctuations in the allelic frequencies due to genetic drift or to habitat changes can occur, particularly in small populations [2]. Compared to large populations, small populations have a much higher possibility of losing genetic diversity due to drift, and thus, they may experience accumulating effects of inbreeding, which results in a relatively low fitness [3,4]. However, the negative effects of inbreeding may be lessened by gene flow [5], while simultaneously decreasing genetic divergence between populations [6]. Thus, knowledge regarding the strength of the evolutionary forces such as gene flow and random genetic drift in the shaping of genetic structure in natural populations has important implications for effective species conservation and management [3,7].

Salmonids, particularly Atlantic salmon (*Salmo salar* L.), show great variation in philopatry (homing) and dispersal (straying) [8], which make them good model systems for the study of such evolutionary processes. They demonstrate a well-known homing ability [9], which combined with the discrete nature of their rearing habitat, results in their structuring into genetically distinct populations e.g., [1,10-12]. However, spatially segregated salmon populations remain interconnected via dispersal [13]. From a practical perspective, the use of temporally replicated samples may assist in the comparison of historical and contemporary patterns of population dispersal, in the detection of changes in population genetic structure and diversity over time, and in the estimation of the level of gene flow between populations, resulting in the selection of proper management units for conservation [14,15]. Moreover, a temporal approach may help to evaluate the genetic consequences of physical and biological environmental changes [16], such as dam building [17], stocking and hatchery supplementations [18,19].

Studies of temporal variation in Atlantic salmon based on neutral markers involve mostly North American [1,10,20,21] and some European salmon populations [18,22-25]. In general, the North American anadromous and freshwater salmon populations have been shown to be temporally stable [1,20,26]. In addition, relative temporal stability has been shown in European anadromous [22,24,27,28] and freshwater [23] salmon. However, temporal genetic instability was also observed in some salmon populations on both sides of the Atlantic due to different reasons, such as variations in the environmental conditions, farm escapees, and pollution [10,25,29].

Atlantic salmon populations of the Western White Sea (particularly of the Kola Peninsula) are generally considered to be stable and less affected by human activities [30]. This region harbours more than 100 salmon rivers, most of which are relatively small with a suitable spawning habitat for 50 to 4000 spawners [31]. Taken together, they have an important effect on salmon reproduction in this region [31]. In addition, there are several larger river systems, such as the Ponoi and Varzuga rivers, which have 30 and 75 thousand ascending salmon spawners every year, respectively [31]. These rivers are also a popular destination for angling tourists. Previous population genetic studies examining single time point samples have revealed a strong subdivision in regional groups with different levels of within group genetic divergence and different patterns of gene flow/genetic drift in each group [32]. In particular, anadromous salmon spawning in the rivers of the Kola Peninsula exhibit a low level of genetic divergence, demonstrating significant isolation by distance [32]. Given the increasing level of human activities in the salmon rivers of the Western White Sea Basin within the past decade e.g., [33,34], the question remains to what degree these activities influence the genetic variation of Atlantic salmon populations in the region.

Here, we characterised the level of temporal genetic variation in 16 anadromous and 2 freshwater Atlantic salmon populations spawning in the rivers of the Western White Sea Basin. We subsequently evaluated the effect of the fishing pressure on the temporal genetic variation in this region. Our specific aim was to determine the level of genetic stability in salmon populations in the Western White Sea, to assess whether the genetic stability was affected by the increasing level of the fishing pressure and, on the basis of the obtained results, to provide management recommendations for conservation.

## Results

A total of 326 alleles were observed across all loci, with locus allele number ranging from 71 in locus Ssa404 to 9 alleles in locus Ssa412. The mean level of genetic diversity across 14 microsatellite loci varied from relatively low in freshwater populations of the Western White Sea Basin (mean *H*_E_ = 0.45; mean *A*_R_ = 3.80) to relatively high in the anadromous populations of the Kola Peninsula (mean *H*_E_ = 0.71; mean *A*_R_ = 9.38) (Table 1). In contrast, the level of population genetic differentiation (*F*_ST_) exhibited the opposite pattern. The highest genetic differentiation was observed among the freshwater populations of the Western White Sea Basin (*F*_ST_ = 0.199), and the lowest was found in the anadromous populations of the Kola Peninsula (*F*_ST_ = 0.012). Five populations (Danilovka 2008, Pyalitsa 2008, Strelna 2008, Indera 2001, and Pisto 1999) were found to deviate from Hardy-Weinberg equilibrium after correcting for multiple (significance) tests. However, these results were treated as negligible due to the occurrences of both heterozygote deficiency and excess at a few loci (see Suppl. online Additional file 1 for details). Similarly, Microchecker indicated the potential occurrence of null alleles at just 10 locus-population combinations (out of 532 tests, Suppl. online Additional file 2), suggesting that the occurrence of common null alleles was unlikely to explain the deviations.

**Table 1:** **Atlantic salmon populations in the basin of the White Sea, sampling locations, year, abbreviation (Abbr.), diversity indices: expected heterozygosity (*H***_**E**_**), observed heterozygosity (*H***_**O**_**), allelic richness (*A***_**R**_**) and inbreeding coefficient ( *f *), the level of fishing pressure ( *Fishing pressure *) and estimated census size ( *Size category *)**

**Population**	**Sampling year**	**Abbr.**	**Coordinates**	**Sample size**	***H***_**E**_	***H***_**O**_	***A***_**R**_	***f***	***Fishing pressure***^***1***^	***Size category***^***2***^
***Anadromous***										
*Kola Peninsula*										
Kachkovka	2008	Kach08	67°24’N40°48’E	66	0.73	0.72	9.52	0.007	2	3
Kachkovka*	2001	Kach01	67°26’N40°56’E	41	0.73	0.70	9.81	0.047	3	3
Ponoi	2008	Ponoi08	67°07’N40°55’E	93	0.73	0.73	9.92	-0.003	1	5
Ponoi	1995	Ponoi95	67°08’N41°04’E	38	0.72	0.73	9.63	-0.008	1	5
Danilovka	2008	Dan08	66°45’N40°59’E	43	0.70	0.68	8.42	0.023	2	1
Danilovka*	2001	Dan01	66°45’N40°58’E	44	0.71	0.72	9.12	-0.009	1	1
Sosnovka	2008	Sos08	66°31’N40°32’E	46	0.71	0.70	9.06	0.040	2	2
Sosnovka	2001	Sos01	66°31’N40°34’E	30	0.74	0.70	9.98	-0.020	1	2
Babya	2008	Baby08	66°25’N40°34’E	41	0.71	0.73	9.27	0.022	2	3
Babya*	2001	Baby01	66°24’N40°08’E	46	0.73	0.71	10.14	0.012	1	3
Likhodeevka	2008	Likh08	66°21’N40°09’E	46	0.72	0.71	9.56	0.012	2	2
Likhodeevka*	2001	Likh01	66°21’N40°08’E	47	0.71	0.71	9.45	0.024	1	2
Pulonga (Kola)	2008	PuK08	66°16’N39°57’E	76	0.70	0.69	9.43	0.010	3	3
Pulonga (Kola)*	2001	PuK01	66°17’N39°56’E	47	0.71	0.70	9.30	-0.038	2	3
Pyalitsa	2008	Plc08	66°11’N39°29’E	26	0.74	0.76	9.65	0.029	3	2
Pyalitsa	2001	Plc01	66°11’N39°30’E	46	0.73	0.71	9.99	0.030	3	2
Chapoma	2008	Cha08	66°07’N38°50’E	49	0.71	0.69	9.73	-0.006	3	3
Chapoma	2001	Cha01	66°06’N38°50’E	42	0.71	0.69	9.36	0.034	4	3
Strelna	2008	Str08	66°04’N38°38’E	63	0.70	0.71	9.92	-0.028	3	3
Strelna	2001	Str01	66°04’N38°38’E	47	0.72	0.69	9.89	0.018	3	3
Chavanga	2008	Chv08	66°09’N37°46’E	41	0.70	0.72	9.34	-0.024	3	3
Chavanga	2001	Chv01	66°07’N37°44’E	49	0.68	0.67	8.05	0.090	4	3
Indera	2008	Ind08	66°14’N37°08’E	60	0.68	0.69	7.99	0.002	3	1
Indera	2001	Ind01	66°14’N37°09’E	45	0.70	0.64	8.73	-0.006	2	1
Varzuga	2008	Var08	66°24’N36°37’E	51	0.71	0.71	9.29	-0.057	4	5
Varzuga	1999	Var99	66°28’N36°28’E	37	0.73	0.73	9.59	-0.100	4	5
*Karelian White Sea*										
Nilma	2005	Nil05	66°29’N33°08’E	32	0.63	0.67	5.52	-0.057	4	1
Nilma*	1999	Nil99	66°29’N33°08’E	42	0.65	0.71	4.92	-0.100	5	1
Pulonga (Karelia)	2005	PuW05	66°18’N33°15’E	56	0.62	0.66	5.32	-0.064	4	1
Pulonga (Karelia)*	1999	PuW99	66°18’N33°15’E	43	0.61	0.63	5.17	-0.026	4	1
Pongoma	2005	Png05	65°17’N34°00’E	41	0.69	0.67	7.69	0.030	3	1
Pongoma*	1999	Png99	65°17’N34°00’E	50	0.68	0.67	7.23	0.013	3	1
***Freshwater***										
Pisto	2005	Pis05	65°21’N30°33’E	39	0.52	0.52	4.48	0.021	5	1
Pisto	1999	Pis99	65°16’N30°33’E	55	0.53	0.52	4.63	-0.002	5	1
Kamennaya	2005	Kam05	64°28’N30°24’E	29	0.39	0.38	3.11	-0.071	3	1
Kamennaya	1999	Kam99	64°28’N30°24’E	57	0.38	0.41	2.98	0.022	3	1

*Data taken from Tonteri et al. [32].

^1^Fishing pressure: 1–no catch in a river; 2-< 15% of spawners caught; 3-16-25% of spawners caught; 4-26-40% of spawners caught; 5-41-70% of spawners caught (A.E. Veselov, A.G. Potutkin–personal observations, Additional file 4: Table S1).

^2^Size category: 1-100-500; 2-500-1000; 3-1000-5000; 4-5000-10000; 5-10000-50000 ascending adults/year [35-38].

### Temporal variation

**Figure 1:**
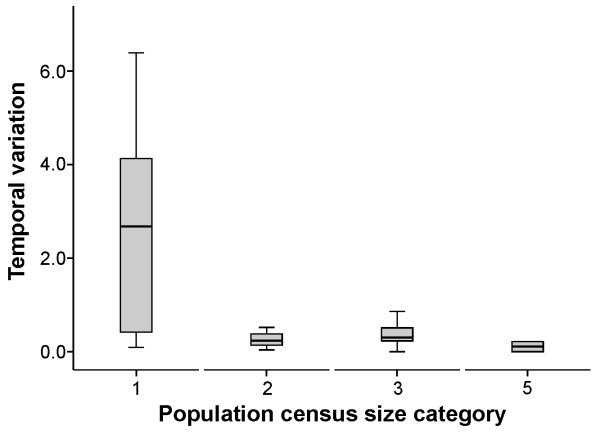
**Box-plot showing the level of temporal variation (%, as revealed by AMOVA) in populations of different population census size category (see Table**1**).** Horizontal line, open rectangle, and whiskers indicate the median, 25th and 75th quartiles, and non-outlier range, respectively.

The population genetic structure of salmon from northwest Russia was characterised by a relatively high temporal stability. Overall, the variation among samples due to the temporal component was nearly 11 times lower than that due to a spatial component as revealed by the AMOVA test (Table 2). For the anadromous populations of the Western White Sea Basin, the overall proportion of temporal *vs.* spatial variation was 1:3, which varied from 1:3.2 for the Kola Peninsula to 1:1.9 for the Karelian White Sea coast salmon. In contrast, for the freshwater populations of the Western White Sea Basin, the variation was nearly 7 times lower due to the higher spatial component (Table 2). In total, a small but significant variation between the temporal samples was detected in eight of the 18 populations of the Western White Sea Basin: seven anadromous (4 from the Kola Peninsula: Danilovka, Chapoma, Strelna, and Chavanga; and 3 from the Karelian White Sea coast: Nilma, Pulonga, and Pongoma) and one freshwater (Pisto). The other 10 populations of the Western White Sea Basin were found to be temporally stable. The level of temporal variation in the anadromous populations of the Kola Peninsula did not exceed 0.86%, whereas the level of temporal variation in the anadromous populations of the Karelian White Sea coast was higher and ranged from 2.68% to 5.14% (Table 2). A small but significant proportion of variation explained by temporal differences (1.19%-6.39%) was observed in all of the Karelian freshwater salmon populations, except for Kamennaya (White Sea Basin) and Hiitola (Lake Ladoga) (Table 2). The relative stability of the genetic composition between the earlier and more recent samples in the Western White Sea Basin was further supported by the low average level of genetic differentiation (*F*_ST_) among the temporal samples within each river (*F*_ST_ = 0.012 ± 0.005), which was nearly 7 times lower than that between the spatial samples (*F*_ST_ = 0.082 ± 0.004). Pairwise genetic differentiation (*F*_ST_) among rivers was not significant in 121 out of 612 tests, all of which involved anadromous populations of the Kola Peninsula (Suppl. online Additional file 3). In general, the level of temporal variation was higher in populations with census sizes of fewer than 500 ascending breeders, in comparison with populations with larger census sizes (Figure 1).

**Table 2:** Analysis of molecular variance (AMOVA) in the temporal samples of Atlantic salmon populations from northwest Russia

**Sample**	**Number of populations**	**Number of groups**	**Percentage of variance**			***P***
			***Within population***	***Among temporal samples***	***Among spatial samples***	
***Whole data (Kola + Karelian White Sea + Karelian freshwater)***	53	26	86.23	1.17	12.60	***
***White Sea basin (Kola + Karelian White sea + Karelian White Sea freshwater)***	36	18	91.83	0.99	7.18	***
***White sea (Kola + White Sea Karelia)***	32	16	96.79	0.80	2.41	***
**Anadromous:**						
*Kola peninsula*	26	13	98.88	0.27	0.85	***
Kachkovka (01 + 08)	2	1	100.00	0.00		ns
Ponoi (95 + 08)	2	1	100.00	0.00		ns
Danilovka (01 + 08)	2	1	99.55	0.45		*
Sosnovka (01 + 08)	2	1	99.48	0.52		ns
Babya (01 + 08)	2	1	99.76	0.24		ns
Lihodeevka (01 + 08)	2	1	99.76	0.24		ns
Pulonga (Kola) (01 + 08)	2	1	99.77	0.23		ns
Pyalitsa (01 + 08)	2	1	99.96	0.04		ns
Chapoma (01 + 08)	2	1	99.49	0.51		*
Strelna (01 + 08)	2	1	99.64	0.36		*
Chavanga (01 + 08)	2	1	99.14	0.86		**
Indera (01 + 08)	2	1	99.91	0.09		ns
Varzuga (99 + 08)	2	1	99.78	0.22		ns
*Karelian White Sea*	6	3	90.45	3.31	6.25	***
Nilma (99 + 05)	2	1	94.86	5.14		***
Pulonga (White sea) (99 + 05)	2	1	97.32	2.68		***
Pongoma (99 + 05)	2	1	96.88	3.12		***
**Freshwater:**						
*White Sea*	4	2	74.33	3.27	22.40	***
Pisto (99 + 05)	2	1	93.61	6.39		***
Kamennaya (99 + 05)	2	1	99.61	0.39		ns
*Lake Onega (Baltic)*	12	5	82.42	2.12	15.46	***
Kumsa (00 + 04)	2	1	95.12	4.88		***
Pyalma (01 + 04)	2	1	98.81	1.19		***
Lizhma (99 + 02 + 04)	3	1	98.44	1.56		**
Shuya (99 + 04)	2	1	98.06	1.94		***
Tuba (01 + 04)	2	1	96.02	3.99		***
*Lake Ladoga (Baltic)*	6	3	91.76	2.16	6.08	***
Hiitola (99 + 06)	2	1	99.46	0.55		ns
Tulema (99 + 06)	2	1	98.46	1.54		***
Sysky (99 + 06)	2	1	96.11	3.89		***

*P < 0.05; **P < 0.01; ***P < 0.001; ns–non significant.

Power analyses tests [39,40] demonstrated that given the number of loci, their polymorphism and the sample sizes used in the study, the probability for detecting of genetic differentiation as low as *F*_ST_ = 0.0005 was over 97%. When *F*_ST_ was set to zero, the proportion of false significances (α) was 4.8%, which is close to the intended value of 5%. Given that the lowest significant *F*_ST_ value observed in our data was 0.0015, it is therefore likely that small but significant *F*_ST_ values detected in our study reflect the true level of genetic differentiation. Furthermore, the null hypothesis of genetic homogeneity was rejected using both χ^2^ and Fisher’s approaches (*P* < 0.0001, both tests) [40,41].

Differences in allelic richness (Wilcoxon signed-rank test *P* < 0.01-0.05) and expected heterozygosity (Wilcoxon signed-rank test *P* < 0.05) per locus between earlier and more recent samples were observed in 6 and 1 populations, respectively, out of 18. However, after sequential Bonferroni correction, none of them remained significant. Correlation of the microsatellite allele frequencies between earlier and more recent samples was significant in all cases and varied from 0.79 to 0.98 (*P* < 0.001). Among anadromous salmon the highest correlation level was observed in populations of the Kola Peninsula (Pearson’s *r* = 0.93-0.97), and the lowest correlation level was found in the populations of the Karelian White Sea coast (Pearson’s *r* = 0.79-0.90). The allelic frequencies of the Karelian freshwater salmon of the Pisto and Kamennaya Rivers demonstrated correlation coefficients of 0.87 and 0.98, respectively.

### Genetic relationships

**Figure 2:**
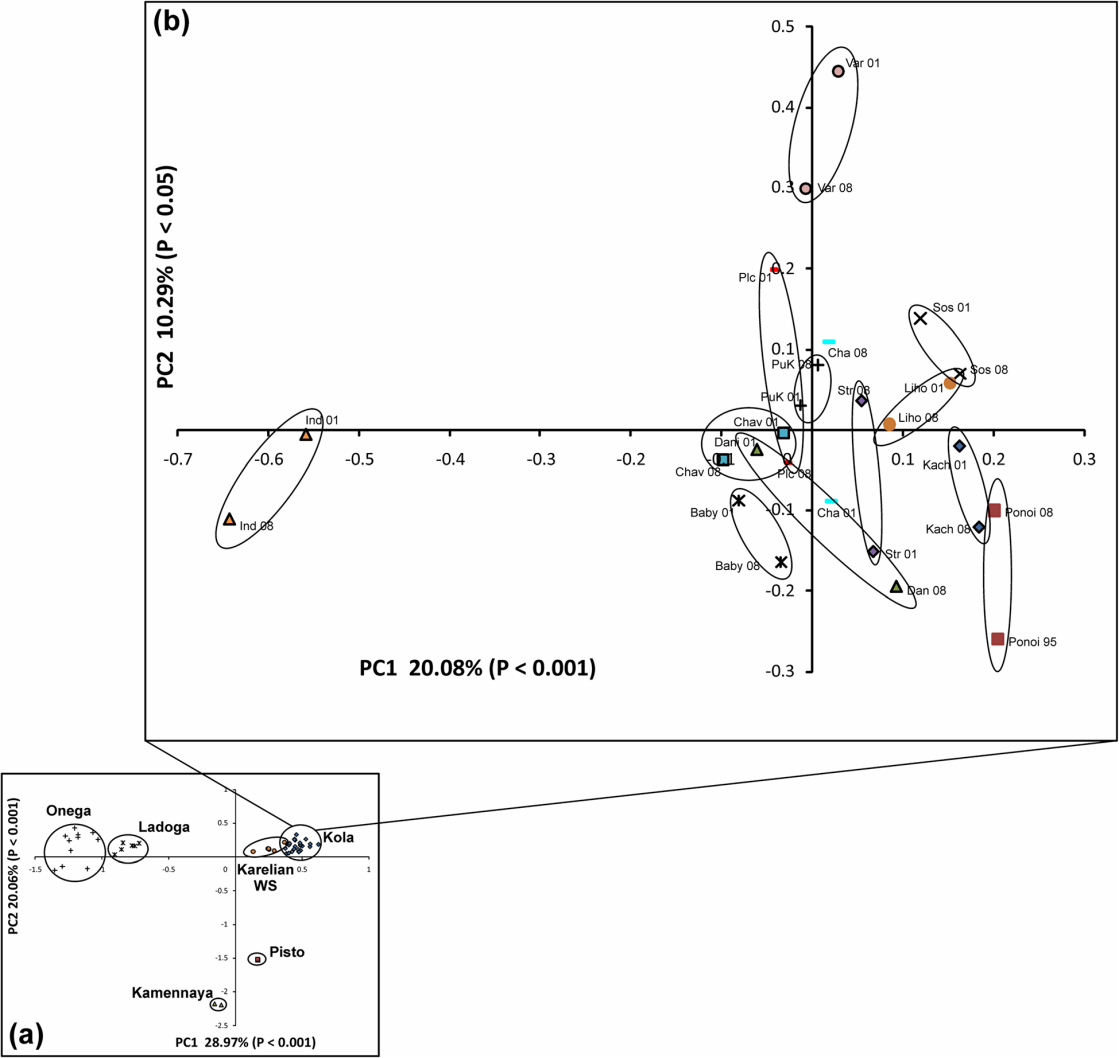
**Principal component analysis (PCA). (a)** Results of the PCA of the Atlantic salmon populations from northwest Russia. Anadromous populations were represented by 2 groups: the Kola Peninsula and Karelian White Sea coast. Freshwater–by the groups of Lakes Ladoga and Onega (Baltic Sea Basin), and populations of the Western White Sea Basin: Kamennaya and Pisto. **(b)** Results of the PCA of the temporal replicates of the anadromous salmon populations of the Kola Peninsula (White Sea Basin).

PCA divided salmon of northwest Russia into four main clusters corresponding to the geographical sampling regions: 1) Kola Peninsula, 2) Karelian White Sea coast, 3) Lake Onega and 4) Lake Ladoga, whereas 5) freshwater populations of the Western White Sea Basin, (Pisto and Kamennaya) were the most distanced from other groups (Figure 2). Here, the first two axes of the principal components captured 49.03% of the total genetic variation. When the populations of the Kola Peninsula were analysed separately, the first two axes represented 30.37% of the total genetic variation. The data points of the temporal replicates were generally located more closely to each other, except for Babya, Indera and Varzuga (Figure 2).

**Figure 3:**
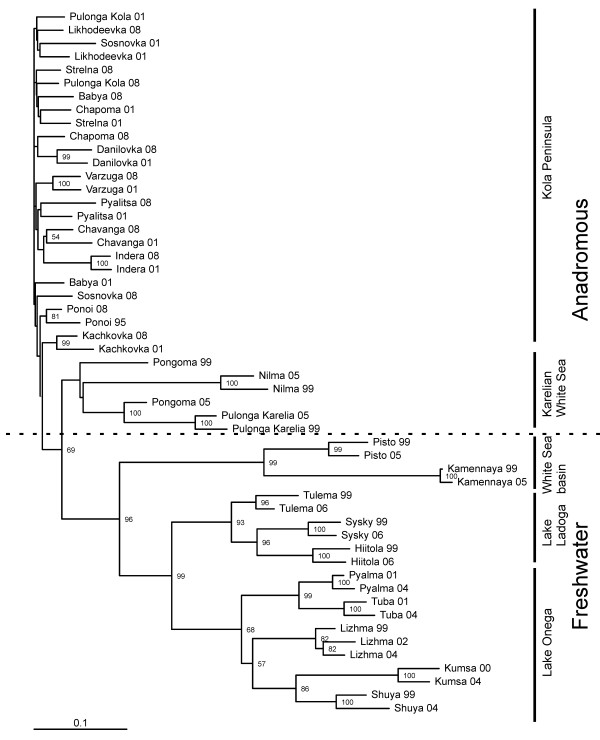
**Neighbour-joining dendrogram based on *D***_**A **_**genetic distances, demonstrating the genetic relationships between the temporally replicated samples of anadromous and freshwater Atlantic salmon populations in the White Sea and Baltic Sea (Lakes Ladoga and Onega) basins.** The number on the nodes indicates the bootstrap values (percentage) obtained after 1000 replicates. Only values >50% are shown.

The salmon populations of northwest Russia also showed a clear subdivision into five groups on the neighbour-joining tree according to their geographical origin (Figure 3). The first cluster was formed by the anadromous populations of the Kola Peninsula, which were separated from the second group, which consisted of the anadromous populations of the Karelian White Sea coast. Anadromous Karelian populations were then grouped with the freshwater salmon of the White Sea Basin (Pisto and Kamennaya), which in turn was grouped with the Karelian freshwater salmon of the Onega and Ladoga Lakes (Figure 3). Nearly half of the temporally replicated anadromous populations of the Kola Peninsula were grouped by the site of origin with a bootstrap support of over 50%. The temporal samples of 3 anadromous and 10 freshwater populations of Karelia generally tended to cluster by the site of origin (except for the Pongoma population) with a bootstrap support that varied between 82% and 100%.

### Isolation by distance

**Figure 4:**
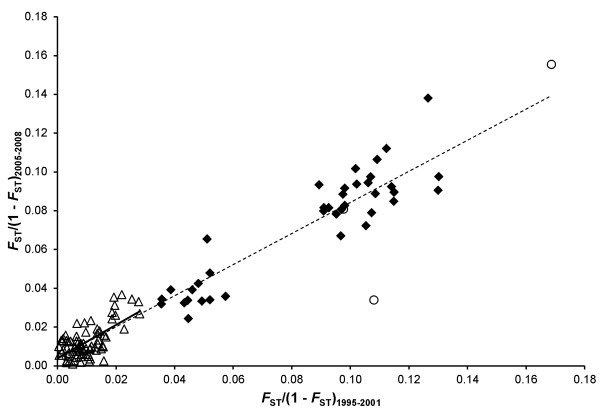
**The relationships between the genetic differentiation among earlier (*F***_**ST**_**/(1-*F***_**ST**_**)**_**1995-2001**_**) and more recent anadromous salmon samples (*F***_**ST**_**/(1-*F***_**ST**_**)**_**2005-2008**_**) of the White Sea Basin (dashed line) (*r***^**2**^ **= 0.92, *P*** **< 0.001) and the Kola Peninsula (solid line) (*r***^**2**^ **= 0.45, *P*** **< 0.001).** The relationships between the Kola, Kola and Karelian White Sea coast, and Karelian White Sea populations are presented as open triangles, closed diamonds and open circles, respectively.

The Mantel test revealed a significant association between the geographic and genetic distance among the anadromous populations of the Western White Sea Basin, which were sampled from 1995-2001 (Mantel’s *r*^2^ = 0.43, *P* < 0.001) and 2005-2008 (Mantel’s *r*^2^ = 0.37, *P* < 0.01) (Additional file 4: Figure S1). This association was weaker but still significant when the test was performed separately in populations from the rivers of the Kola Peninsula (Mantel’s *r*^2^_1995-20001_ = 0.15, *P*_1995-2001_ < 0.05; Mantel’s *r*^2^_2005-2008_ = 0.20, *P*_2005-2008_ < 0.05, Additional file 4: Figure S1). The genetic differentiation pattern was also consistent among the earlier and more recent samples (Mantel’s test *F*_ST1995-2001_*vs*. *F*_ST2005-2008_, *r*^2^ = 0.92, *P* < 0.001) (Figure 4), which provided additional evidence of the relative temporal stability of the Atlantic salmon populations in the Western White Sea Basin.

### Effects of fishing pressure

**Figure 5:**
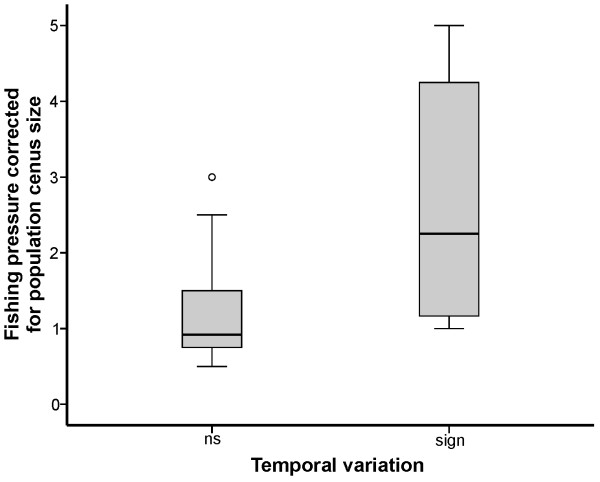
**Box-plot showing the significant difference in fishing pressure (corrected for population census size) on populations with significant (sign, *P*** **< 0.05, *n*** **= 8) and non-significant (ns, *n*** **= 10) genetic variation between the temporal samples (non-parametric Mann–Whitney U-test, *P*** **< 0.05).** Horizontal line, open rectangle, whiskers, and open circle indicate the median, 25th and 75th quartiles, non-outlier range and an outlier, respectively. Fishing pressure was corrected for population census size by dividing the mean fishing pressure for the two sampling years by the estimated census size class of each population.

**Figure 6:**
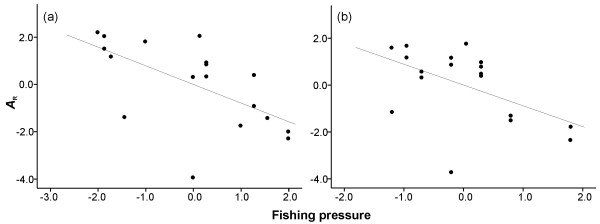
**Partial residual scatter plot demonstrating the significant negative correlation between allelic richness (*A***_**R**_**) and fishing pressure on Atlantic salmon populations in the Western White Sea region in (a) 1995-2001 and (b) in 2005-2008, which was controlled for the estimated census size (Pearson’s partial correlation *r***_**1995-2001**_ **=-0.60, *P***_**1995-2001**_ **< 0.01 and *r***_**2005-2008**_ **=-0.51, *P***_**2005-2008**_ **< 0.05, respectively).**

A negative association between the level of temporal stability of allele frequencies and fishing pressure was also evident. The fishing pressure index (Additional file 4: Table S1) was significantly higher in populations exhibiting a significant change in allele frequencies over time compared to populations whose allele frequencies did not significantly change (non-parametric Mann–Whitney U-test, *P* < 0.05, Figure 5). Similarly, temporal stability tended to be lower in populations of the Kola Peninsula experiencing higher fishing pressure, however this trend was non-significant (non-parametric Mann–Whitney U-test, *P* = 0.12, Additional file 4: Figure S2). Furthermore, when the effect of the estimated census size of a population was controlled, there was a significant negative partial correlation between allelic richness and fishing pressure (Pearson’s partial correlation *r*_1995-2001_ =-0.60, *P*_1995-2001_ < 0.01 and *r*_2005-2008_ =-0.51, *P*_2005-2008_ < 0.05, Figure 6). The trend of partial correlation of *A*_R_ and fishing pressure remained negative, albeit non-significant, when tested separately for 13 populations of the Kola Peninsula (Pearson’s partial correlation *r*_1995-2001_ =-0.40, *P*_1995-2001_ = 0.20 and *r*_2005-2008_ =-0.17, *P*_2005-2008_ = 0.61).

## Discussion

### Temporal variation

The level of temporal stability varied among the Atlantic salmon populations of the Western White Sea Basin. A small but significant proportion of variation explained by the temporal component was observed among 8 populations (7 anadromous and 1 freshwater), whereas the genetic composition of 10 populations was temporally stable. The lowest proportion of the temporal component observed in the anadromous populations of the Kola Peninsula (0.00%-0.86%) was most likely attributed to their higher census and effective population sizes compared to salmon from the Karelian White Sea coast [42], in which this component was considerably higher (2.68%-5.14%). Indeed, we observed higher level of temporal variation in populations of smaller census sizes and are thus more affected by genetic drift. Whereas genetic drift is compensated by gene flow in Kola Peninsula populations, the significantly higher level of temporal variation in salmon populations from the Karelian White Sea coast suggests a stronger role of genetic drift in shaping their genetic structure [32]. The more stable genetic structuring of the Kola Peninsula salmon over time was supported by non-significant genetic differentiation (*F*_ST_) and higher correlations of allele frequencies (Pearson’s *r* = 0.93-0.97, *P* < 0.001) between the temporal samples in comparison with the Karelian White Sea coast anadromous populations (Pearson’s *r* = 0.79-0.90, *P* < 0.001). Overall, the temporal variation within the sampling sites observed in the Western White Sea Basin over a period of approximately 1-1.5 generations (for the River Ponoi–approximately 2.5 generations) was relatively minor compared to the spatial differences between the two. This pattern is typical for salmonids, and several studies have demonstrated the temporal stability of the genetic structure of anadromous and freshwater populations e.g., [1,7,22,26,28,43,44].

However, the effects of migration and genetic drift in shaping the genetic structure of salmon populations in this region may be also influenced by environmental and, in particular, anthropogenic factors. We observed a significant decrease in the genetic diversity of salmon populations with increasing levels of fishing pressure (Pearson’s partial correlation *r*_1995-2001_ =-0.60, *P*_1995-2001_ < 0.01 and *r*_2005-2008_ =-0.51, *P*_2005-2008_ < 0.05). These associations were more clearly manifested in populations with small census and effective population sizes, although the effect was observed across census size classes. Moreover, populations demonstrating a significant level of temporal variation in the genetic composition were more affected by fishing pressure compared to the populations with non-significant genetic changes over time (non-parametric Mann–Whitney U-test, *P* < 0.05). These associations remained negative, albeit non-significant, when including only populations of the Kola Peninsula, indicating that populations of the western White Sea basin are important contributors to this trend. The observed associations between human activities and genetic variation support the notion of a higher fishing pressure on the populations of the Karelian White Sea coast than on the populations of the Kola Peninsula [33,45]. Such differences may be attributed to the rivers of the Karelian White Sea coast, which have better road approaches, while most of the salmon rivers of the Kola Peninsula are located in more remote areas, which are only accessible by helicopters or boats [33,45]. Moreover, the human population density in the districts of the Kola Peninsula (Tersky and Lovozersky) with the most productive salmon rivers, such as Ponoi and Varzuga, was approximately 0.26/km^2^. In contrast, in the Karelian White Sea coast, the human population density was 7 times higher at 1.92/km^2^ (http://www.gks.ru).

Interestingly, the temporal changes in the genetic composition of a salmon population might be attributed to other factors, such as stocking supplementations e.g., [18] or farm escapees e.g., [25,29]. However, these scenarios seem less likely in the Western White Sea Basin. Despite regular farmed salmon releases in some of the rivers of the Southern White Sea coast (Keret’ and Luvenga), genetic introgression due to straying individuals in other Karelian rivers appears to be minor. First, the Kandalaksha farm only works with the Luvenga population, and Kem’ and Vyg have both propagated only the Keret’ population for more than 30 years [46]. Second, the southern part of the White Sea is characterised by an irregularity of its coastline and complex currents. The other two farms, Taibolskiy and Umbskiy, are located far away from the sampling sites, use the local salmon spawners and release smolts into the river of origin. Thus, we consider it unlikely that farmed fish influenced the genetic composition of the wild salmon in this region.

Importantly, increased fishing pressure was recently observed in most of the rivers of the region (A.E. Veselov, unpublished data). However, due to compensation of sufficiently large census and effective population sizes of most Atlantic salmon populations in the Western White Sea Basin, the effects of such changes may take a larger number of generations to be detectable [47,48]. Considering that population stability increases with increasing population size [49], we observed highly significant changes in the genetic composition only in a few populations, most of which have estimated census sizes of fewer than 500 ascending adults (salmon of the Karelian White Sea coast). Moreover, small census and effective population sizes combined with a relatively high fishing pressure make these salmon populations more vulnerable to environmental and climate changes. Therefore, given the signs of genetic instability observed over a relatively short time period (1-1.5 generations), future genetic monitoring of these Karelian White Sea populations is recommended. Consequently, the analyses of more temporal replicates, which ideally include older samples, together with historical ecological observations may provide a broader view regarding the potential anthropogenic and environmental effects on salmon populations in the Western White Sea Basin.

### Genetic substructure

The observed pattern of a strong subdivision of Atlantic salmon populations from the White Sea Basin into four large groups was consistent with earlier studies in this region [23,50-52]. While the temporal replicates of freshwater and most of the Karelian White Sea coast anadromous populations (except for Pongoma) tended to cluster together, only half of the populations of the Kola Peninsula demonstrated a similar trend. Moreover, on the PCA plot, the temporal samples of only 3 populations of the Kola Peninsula were separately grouped. This pattern might indicate a higher relative role of migration in the shaping of genetic structure in salmon populations of the Kola Peninsula compared to the anadromous populations of the Karelian White Sea coast. This was also supported by a low level of between-river genetic differentiation, which was not significant in 20% of the tests and the IBD pattern among the populations of the Kola Peninsula, which was significant in two temporal replicates (1995-2001 and 2008). However, in both cases, the regression slope was low (*r*^2^ = 0.15-0.17), providing support for gene flow overpowering genetic drift (case II, [53]). This may also explain the lack of resolution on the neighbour-joining tree among Kola Peninsula populations combined with the close geographical proximity of the sampling locations in the area compared to other areas in the study.

Overall, a significant isolation by distance signal among the anadromous populations in the Western White Sea Basin was consistent with previous observations reported in other studies on salmonid populations and particularly in anadromous Atlantic salmon populations [32,54-58]. Moreover, the consistency of the IBD pattern among earlier and more recent samples of anadromous salmon has provided additional evidence of the relative temporal stability of the population genetic structure in this region. Importantly, the observed IBD pattern indicates that gene flow and genetic drift influence the regional population structure differently depending on the scale: at shorter geographic distances, gene flow is more effective, whereas the genetic drift has a greater influence at greater distances of geographical separation (case IV, [53]). This was consistent with the observations of Tonteri et al. [32], suggesting that Atlantic salmon of the White and Barents Seas were still undergoing a transitory phase towards equilibrium between gene flow and drift.

## Conclusions

In general, a higher level of temporal stability of Atlantic salmon population structure was observed in the relatively pristine area of the Kola Peninsula, whereas populations inhabiting the rivers located in more populated area (Republic of Karelia) demonstrated greater temporal variation. Considering the negative influence of fishing pressure on the genetic structure and diversity of salmon observed in this region, conservation measures should be a key component of stock management strategies. Given that the temporal variation was more clearly manifested in populations with small census and effective population sizes the protection and genetic monitoring of such populations is recommended, and particular attention should be paid to the Karelian rivers.

## Methods

### Samples and microsatellite analysis

**Figure 7:**
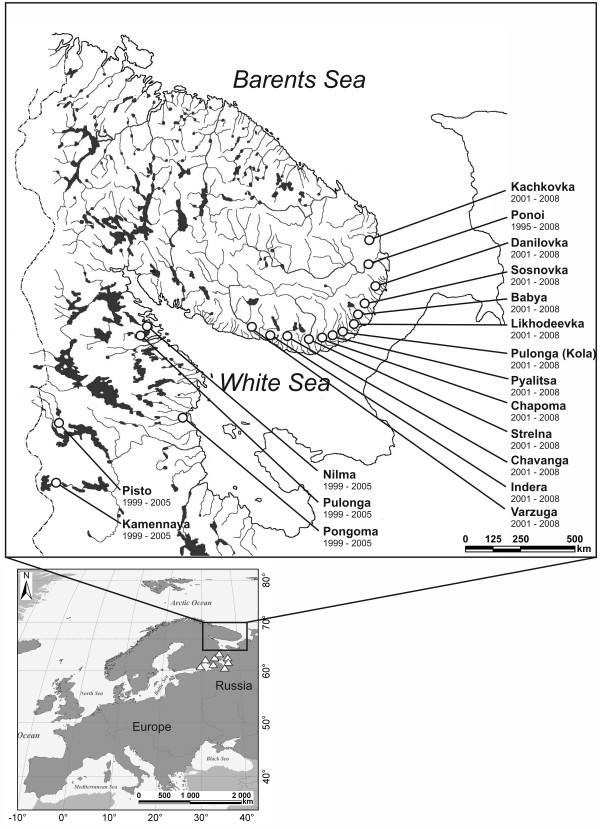
**Map indicating the sampling locations of the studied populations.** The sampling locations of freshwater populations [23] for comparative analyses are presented as triangles on the insert map.

The samples (n = 1344, fin clips) were collected from Atlantic salmon 1+ - 3+ parr between 1995-2001 and 2005-2008 in 16 salmon rivers distributed along the Kola Peninsula and the Karelian White Sea coast as well as in 2 freshwater rivers in Karelia (the White Sea Basin, Figure 7, Table 1). The average time between sampling within a site was 7.2 years (range 6-13), i.e., approximately 1.5 generations. Samples from each location were electrofished along a river stretch that normally spanned 100-200 m^2^, and samples from different time points were collected from within 4 km of the previous sampling location, except for Pisto (12 km) and Varzuga (10 km). Protocols for DNA extraction, PCR and 14 microsatellite loci genotyping have been previously described by Tonteri et al. [32]. The microsatellite data from a single time point for eight anadromous populations (sampling year: 1999-2001) were obtained from Tonteri et al. [32] (Table 1). Temporal microsatellite data for eight additional freshwater Karelian populations (Lakes Ladoga and Onega) were obtained from Ozerov et al. [23] and were included in the study for comparative purposes. Analyses of the microsatellite data were performed in the same laboratory, and the same microsatellite allele bins and scoring protocols were performed as previously described by Tonteri et al. [32]. In total, the population genetic data for 18 temporally replicated salmon populations from the Western White Sea Basin were available for this study.

### Data analysis

The basic descriptive statistics for each population and locus (allelic richness, expected and observed heterozygosity) were obtained using Fstat 2.93 [59]. In addition, the same software was used to calculate Weir and Cockerham’s [60] within-population inbreeding coefficients (*f*) and between-population fixation indices (*θ*). The simulated Fisher’s exact test [61] implemented in GenePop 4.0 [62] was used to estimate deviations from the Hardy-Weinberg equilibrium for loci and populations. The potential presence of genotyping errors, such as large allele dropouts and null alleles, was examined using the Brookfield 1 estimator [63] implemented in the computer software package Micro-Checker 2.2.3 [64].

### Temporal stability of population genetic structure

The significance of genetic differentiation between populations (Weir and Cockerham’s [60] pairwise *F*_ST_) was obtained after 12600 permutations using Fstat 2.93 [59]. The relationships between the temporal and spatial samples were then visualised on the basis of principal component analysis performed using Pca-Gen 1.2 [65], in which the statistical significance of the axes was obtained after 2000 randomisations. In addition, the between population *D*_A_ genetic distances [66] were calculated and a neighbour-joining tree [67] was constructed using Populations 1.2.3 [68]. The tree robustness was tested with 1000 bootstraps among loci. Furthermore, the stability of the population structure was also examined by comparing the temporal variation within rivers with the spatial variation among rivers by applying a two-level hierarchical analysis of molecular variance (AMOVA) using Arlequin 3.5 [69]. The separation among anadromous populations was made between 1) Kola Peninsula and 2) Karelian coast of the White Sea. The freshwater salmon were divided according to their basin of origin: 1) freshwater populations of the White Sea Basin, 2) Lake Onega (Baltic Sea) and 3) Lake Ladoga (Baltic Sea). In addition, the temporal stability of the allelic frequencies was quantified by computing Pearson’s correlation coefficients between the frequencies for each allele observed in two temporal samples for each population. Differences in the estimates of the expected heterozygosity (*H*_E_) and allelic richness (*A*_R_) per locus between temporal samples of each population were assessed using a non-parametric Wilcoxon signed-rank test.

POWSIM v4.1 [39,40] was used to estimate whether the dataset used for genetic population analysis provided enough statistical power for detecting significant genetic differentiation. We simulated scenarios assuming different local population sizes, including our actual sample sizes. Simulations were run using various combinations of *N*_*e*_ and *t* (where *N*_*e*_ is the effective population size and *t* is the time since divergence, respectively), leading to *F*_ST_ as low as 0.0005. The most polymorphic locus Ssa404 was excluded from the analysis, due to software limitations allowing ≤ 50 alleles per locus. Significance estimates were based on 1000 independent simulations. We also estimated the α error (type I) by performing a simulation of no divergence among samples (i.e. setting *t* = 0 that leads a value of *F*_ST_ = 0). Additionally, the hypothesis of no difference at any locus using the actual genotype data was tested applying χ^2^ and Fisher’s approaches implemented in the computer software package CHIFISH 1.3 [40,41]).

The level of fishing pressure (Table 1) was estimated on a 1-5 scale as the proportion of spawning individuals harvested on the basis of the fishing control authority reports, in-river exploitation (i.e., presence of villages, angling touristic camps, poaching), and personal observations (Additional file 4: Table S1). Census size of Atlantic salmon populations in large rivers (Ponoi and Varzuga) was estimated based on the count of ascending adults using fish-count traps. In small rivers (Indera, Pulonga, and Pyalitsa) census size was estimated as the mean of total count of seaward running smolts and by smolt tagging and count of returned spawners (Chapoma and Strelna) [35-38]. For other streams the census sizes were estimated as in Power [70] on the basis of spawning and nursery habitat area, its quality, and densities of salmon juveniles of different age classes counted using electrofishing. The estimates are averaged over ~ 15 year period of monitoring of the salmon rivers included in the present study.

The effect of a fishing pressure on the genetic diversity of salmon populations in the region was tested using the SAS ver. 9.2 program (SAS Institute, Inc., Cary, NC, USA), which applied a partial correlation between the allelic richness and the estimated level of fishing pressure while controlling for the estimated census size. In addition, the relationship between the level of temporal stability of the allelic frequencies in a population and the fishing pressure (corrected for the estimated census size of a population) was examined by applying a non-parametric Mann–Whitney U-test. Mantel’s test was implemented using GenAlEx 6 [71] and was used to clarify if the geographic distance was associated with the genetic divergence among populations (i.e., if the isolation by distance (IBD) signal was evident). The analysis was performed for only 16 anadromous populations of the Western White Sea Basin and was performed separately for the Kola Peninsula salmon (13 populations) with a statistical significance being accessed with 9999 random permutations. The geographic distances among the populations were calculated as the shortest water distances between the sampling sites, and the pairwise genetic divergence was estimated as *F*_ST_/(1-*F*_ST_). To confirm the consistency of the migration pattern over time, the test was performed separately for earlier (1995-2001) and more recent (2005-2008) samples. In addition, Mantel’s test (9999 random permutations) was performed to compare the consistency of the pairwise *F*_ST_ values between the earlier and recent samples of 16 anadromous populations in the Western White Sea Basin.

## Competing interests

The authors declare that they have no competing interests.

## Authors’ contributions

MO performed the laboratory and data analysis, contributed to the study design and drafted the manuscript. CP designed the study, significantly contributed to the data analysis and the writing of the manuscript and assisted with collection of biological samples. AV and JL took part in designing the study, contributed to revisions of the manuscript and collected biological samples. All authors read and approved the final manuscript.

## Supplementary Material

Additional file 1 **Microsatellite diversity indices of the 32 studied Atlantic salmon populations and the results of the Hardy-Weinberg equilibrium test.** The year of sampling is indicated after population code. *N*–number of individuals; *A*–average number of alleles in a population; *H*_O_–observed heterozygosity; *H*_E_–expected heterozygosity; HWE–the *P* value of the Hardy-Weinberg equilibrium test. The *P* values over all populations and loci have been corrected for multiple significant tests. Click here for file

Additional file 2 Estimation of null allele frequencies (Brookfield 1) across loci and populations. Click here for file

Additional file 4 Table S1 Estimated census size and the evaluation of anthropogenic pressure in the rives of the Western White Sea basin. **Figure S1.** The relationships between genetic (*F*_ST_/(1-*F*_ST_) and geographic distances for the anadromous populations of the White Sea Basin (dashed line) (*r*^2^_1995-2001_ = 0.39; *r*^2^_2005-2008_ = 0.35, *P* < 0.001 both tests); and the Kola Peninsula (solid line) (*r*^2^_1995-2001_ = 0.15; *r*^2^_2005-2008_ = 0.17, *P* < 0.05 both tests), sampled in (a) 1995-2001 and (b) 2005-2008. The relationships between Kola, Kola and Karelian White Sea coast, and Karelian White Sea populations are presented as open triangles, closed diamonds and open cirlces, respectively. **Figure S2.** Box-plot showing the difference in fishing pressure (corrected for population census size) on populations of the Kola Peninsula with significant (sign, *P* < 0.05, *n* = 4) and non-significant (ns, *n* = 9) genetic variation between the temporal samples (non-parametric Mann–Whitney U-test, *P* = 0.12). Horizontal line, open rectangle, whiskers, and open circle indicate the median, 25th and 75th quartiles, non-outlier range and an outlier, respectively. Fishing pressure was corrected for population census size by dividing the mean fishing pressure for the two sampling years by the estimated census size class of each population. Click here for file

Additional file 3 **Pairwise genetic distances (*F***_**ST**_**) among Atlantic salmon populations in the basin of the White Sea based on microsatellite data as measured with *F***_**ST**_**.** Salmon populations of the Kola Peninsula are shaded in grey. Click here for file
