# Supplementary material for: Temporal variation of genetic composition in Atlantic salmon populations from the Western White Sea Basin: influence of anthropogenic factors?
Source: BMC Genet. 2013 Sep 23;14:88. doi: 10.1186/1471-2156-14-88 (PMC3852729; doi:10.1186/1471-2156-14-88)
Supplement: Additional file 4 Table S1 — Estimated census size and the evaluation of anthropogenic pressure in the rives of the Western White Sea basin. Figure S1. The relationships between genetic (FST/(1-FST) and geographic distances for the anadromous populations of the White Sea Basin (dashed line) (r21995-2001 = 0.39; r22005-2008 = 0.35, P < 0.001 both tests); and the Kola Peninsula (solid line) (r21995-2001 = 0.15; r22005-2008 = 0.17, P < 0.05 both tests), sampled in (a) 1995-2001 and (b) 2005-2008. The relationships between Kola, Kola and Karelian White Sea coast, and Karelian White Sea populations are presented as open triangles, closed diamonds and open cirlces, respectively. Figure S2. Box-plot showing the difference in fishing pressure (corrected for population census size) on populations of the Kola Peninsula with significant (sign, P < 0.05, n = 4) and non-significant (ns, n = 9) genetic variation between the temporal samples (non-parametric Mann–Whitney U-test, P = 0.12). Horizontal line, open rectangle, whiskers, and open circle indicate the median, 25th and 75th quartiles, non-outlier range and an outlier, respectively. Fishing pressure was corrected for population census size by dividing the mean fishing pressure for the two sampling years by the estimated census size class of each population. [file 1471-2156-14-88-S4.doc]

Table S1. Estimated census size and the evaluation of anthropogenic pressure in the rives of the Western White Sea basin. The ‘overall pressure’ index was used in analyses.

| **River** | **Year** | **Estimated census size** | **Level of poaching** | **Accessibility of the river** | **Human habitation level** | **Commercial and recreational fishing pressure** | **Level of conservation measures** | **Overall pressure due to fishing** |  |
| --- | --- | --- | --- | --- | --- | --- | --- | --- | --- |
| *Kola Peninsula* | |  |  |  |  |  |  |  |  |
| Kachkovka | 2008 | 1100 | low | medium | low | medium | medium | **2** |  |
| Kachkovka | 2001 | -”- | medium | medium | low | low | low | **3** |  |
| Ponoi | 2008 | 30000 | low | medium | medium | medium | high | **1** |  |
| Ponoi | 1995 | -”- | low | medium | medium | medium | high | **1** |  |
| Danilovka | 2008 | 150 | medium | low | low | low | low | **2** |  |
| Danilovka | 2001 | -”- | low | low | low | low | low | **1** |  |
| Sosnovka | 2008 | 650 | medium | low | low | low | low | **2** |  |
| Sosnovka | 2001 | -”- | low | low | low | low | low | **1** |  |
| Babya | 2008 | 1000 | medium | low | low | low | low | **2** |  |
| Babya | 2001 | -”- | low | low | low | low | low | **1** |  |
| Likhodeevka | 2008 | 500 | medium | low | low | low | low | **2** |  |
| Likhodeevka | 2001 | -”- | low | low | low | low | low | **1** |  |
| Pulonga (Kola) | 2008 | 1500 | medium | medium | low | low | low | **3** |  |
| Pulonga (Kola) | 2001 | -”- | medium | low | low | low | low | **2** |  |
| Pyalitsa | 2008 | 650 | medium | medium | medium | low | low | **3** |  |
| Pyalitsa | 2001 | -”- | medium | low | medium | low | low | **3** |  |
| Chapoma | 2008 | 1000 | medium | medium | high | medium | high | **3** |  |
| Chapoma | 2001 | -”- | high | low | high | medium | low | **4** |  |
| Strelna | 2008 | 4000 | medium | low | low | medium | medium | **3** |  |
| Strelna | 2001 | -”- | low | low | low | medium | medium | **3** |  |
| Chavanga | 2008 | 4000 | medium | medium | high | medium | high | **3** |  |
| Chavanga | 2001 | -”- | high | low | high | medium | low | **4** |  |
| Indera | 2008 | 400 | medium | medium | low | low | low | **3** |  |
| Indera | 2001 | -”- | low | low | low | low | low | **2** |  |
| Varzuga | 2008 | 75000 | medium | high | high | high | high | **4** |  |
| Varzuga | 1999 | -”- | medium | high | high | high | high | **4** |  |
| *Karelian coast* | |  |  |  |  |  |  |  |  |
| Nilma | 2005 | 50 | high | high | high | low | low | **5** |  |
| Nilma | 1999 | -”- | high | high | high | low | low | **5** |  |
| Pulonga (White Sea) | 2005 | 300 | medium | high | high | low | low | **4** |  |
| Pulonga (White Sea) | 1999 | -”- | medium | high | high | low | low | **4** |  |
| Pongoma | 2005 | 400 | medium | medium | high | low | low | **3** |  |
| Pongoma | 1999 | -”- | medium | medium | high | low | low | **3** |  |
| *Landlocked (White Sea basin)* | | |  |  |  |  |  |  |  |
| Pistojoki | 2005 | 50 | high | high | high | medium | low | **4** |  |
| Pistojoki | 1999 | -”- | medium | high | high | low | low | **3** |  |
| Kamennaya | 2005 | 100 | low | low | medium | low | high | **1** |  |
| Kamennaya | 1999 | -”- | low | low | medium | low | high | **1** |  |


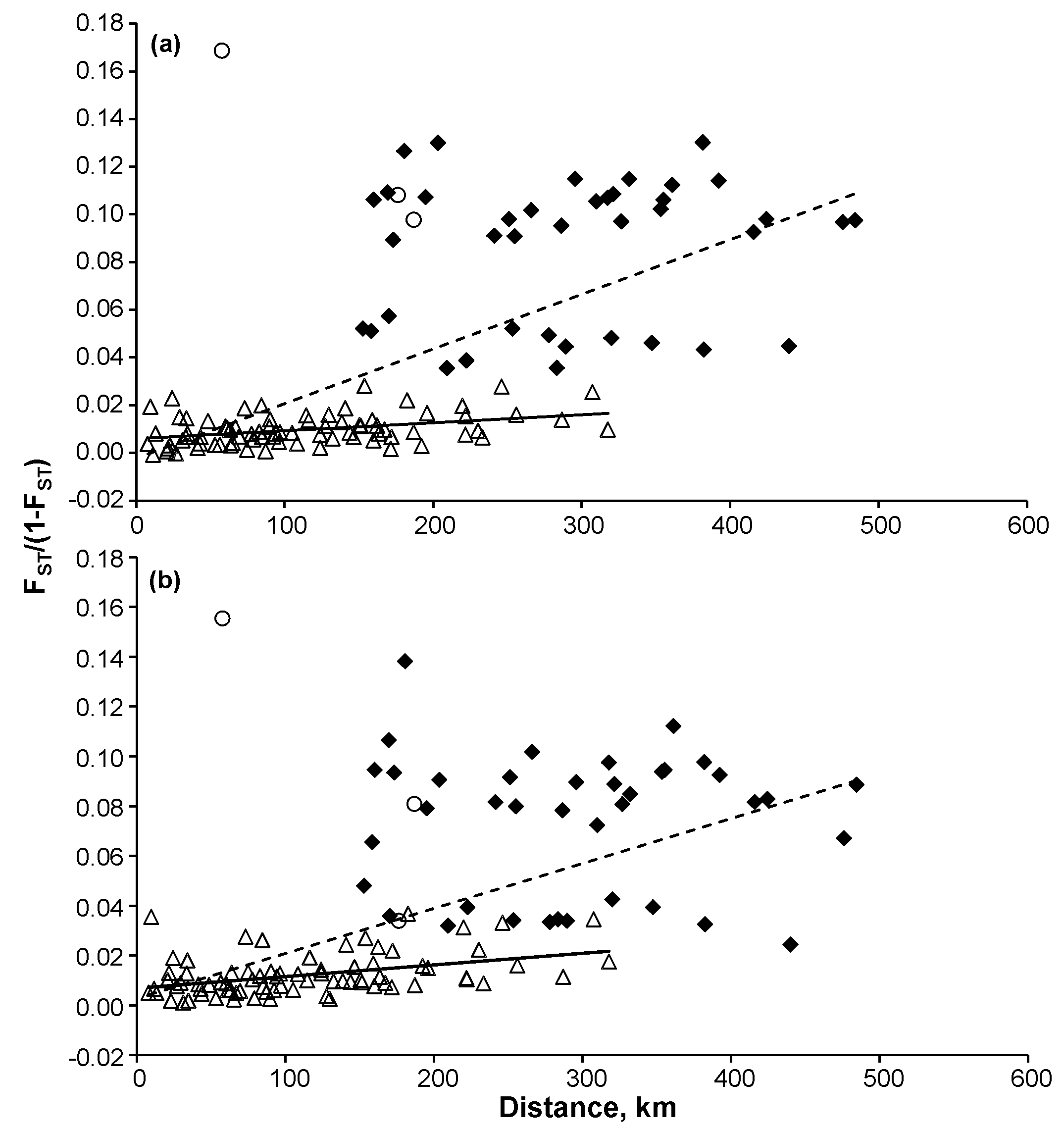


Fig. S1. The relationships between genetic (*F*_ST_/(1-*F*_ST_) and geographic distances for the anadromous populations of the White Sea basin (dashed line) (*r*^2^_1995-2001_= 0.39; *r*^2^_2005-2008_ = 0.35, *P* < 0.001 both tests); and the Kola Peninsula (solid line) (*r*^2^_1995-2001_ = 0.15; *r*^2^_2005-2008_ = 0.17, *P* < 0.05 both tests), sampled in (a) 1995 – 2001 and (b) 2005 – 2008. The relationships between Kola, Kola and Karelian White Sea coast, and Karelian White Sea populations are presented as open triangles, closed diamonds and open cirlces, respectively.

**
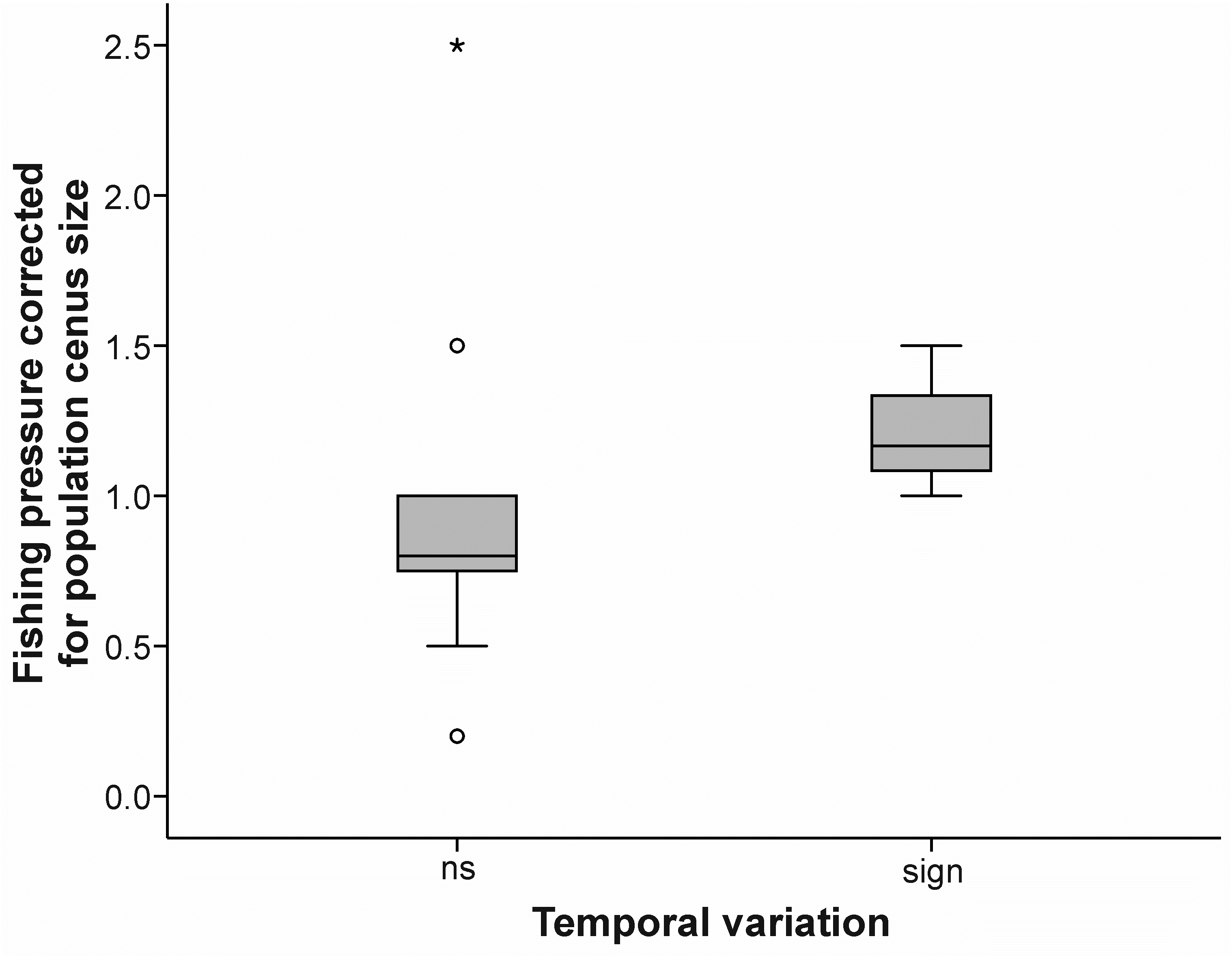
**

Figure S2. Box-plot showing the difference in fishing pressure (corrected for population census size) on populations of the Kola Peninsula with significant (sign, *P* < 0.05, *n* = 4) and non-significant (ns, *n* = 9) genetic variation between the temporal samples (non-parametric Mann-Whitney U-test, *P* = 0.12). Horizontal line, open rectangle, whiskers, and open circle indicate the median, 25th and 75th quartiles, non-outlier range and an outlier, respectively. The correction of fishing pressure for population census size was estimated by dividing mean fishing pressure for 2 sampling years of each population by estimated census size class.
